# Parathyroid Hormone-Related Peptide Elicits Peripheral TRPV1-dependent Mechanical Hypersensitivity

**DOI:** 10.3389/fncel.2018.00038

**Published:** 2018-02-15

**Authors:** Andrew J. Shepherd, Aaron D. Mickle, Suraj Kadunganattil, Hongzhen Hu, Durga P. Mohapatra

**Affiliations:** ^1^Department of Pharmacology, Roy J. and Lucille A. Carver College of Medicine, University of Iowa, Iowa City, IA, United States; ^2^Washington University Pain Center, Department of Anesthesiology, Washington University School of Medicine in St. Louis, St. Louis, MO, United States; ^3^Center for the Study of Itch, Department of Anesthesiology, Washington University School of Medicine in St. Louis, St. Louis, MO, United States; ^4^Center for Investigation on Membrane Excitable Diseases, Washington University School of Medicine in St. Louis, St. Louis, MO, United States

**Keywords:** PTHrP, TRPV1, TRPA1, TRPV4, pain, mechanical pain, cancer pain

## Abstract

Bone metastasis in breast, prostate and lung cancers often leads to chronic pain, which is poorly managed by existing analgesics. The neurobiological mechanisms that underlie chronic pain associated with bone-metastasized cancers are not well understood, but sensitization of peripheral nociceptors by tumor microenvironment factors has been demonstrated to be important. Parathyroid hormone-related peptide (PTHrP) is highly expressed in bone-metastasized breast and prostate cancers, and is critical to growth and proliferation of these tumors in the bone tumor microenvironment. Previous studies have suggested that PTHrP could sensitize nociceptive sensory neurons, resulting in peripheral pain hypersensitivity. In this study, we found that PTHrP induces both heat and mechanical hypersensitivity, that are dependent on the pain-transducing *t*ransient *r*eceptor *p*otential channel family *v*anilloid, member-1 (TRPV1), but not the mechano-transducing TRPV4 and TRPA1 ion channels. Functional ratiometric Ca^2+^ imaging and voltage-clamp electrophysiological analysis of cultured mouse DRG neurons show significant potentiation of TRPV1, but not TRPA1 or TRPV4 channel activation by PTHrP. Interestingly, PTHrP exposure led to the slow and sustained activation of TRPV1, in the absence of any exogenous channel agonist, and is dependent on the expression of the type-1 parathyroid hormone receptor (PTH1), as well as on downstream phosphorylation of the channel by protein kinase C (PKC). Accordingly, local administration of specific small-molecule antagonists of TRPV1 to mouse hindpaws after the development of PTHrP-induced mechanical hypersensitivity led to its significant attenuation. Collectively, our findings suggest that PTHrP/PTH1-mediated flow activation of TRPV1 channel contributes at least in part to the development and maintenance of peripheral mechanical pain hypersensitivity, and could therefore constitute a mechanism for nociceptor sensitization in the context of metastatic bone cancer pain.

## Introduciton

Advanced breast and prostate cancer frequently metastasize to bones and growth of these metastatic tumors is often associated with severe pain and discomfort (Roodman, 2004; Papachristou et al., 2012; Mantyh, 2013; Rucci and Angelucci, 2014; Schmidt, 2014). This type of pain is often undermanaged with existing analgesics, due to the development of tolerance and dose limiting side effects of traditional opioids (Mantyh, 2013; Schmidt, 2014; Lucchesi et al., 2017). While the precise mechanisms underlying metastatic bone cancer pain are poorly understood, it is thought to be initiated in part by sensitization of peripheral nociceptors innervating the site of bone tumor growth (Schmidt, 2014). Multiple studies have shown that cytokines, chemokines, growth factors and other peptides released into the tumor microenvironment sensitize nociceptive fibers (Constantin et al., 2008; Schweizerhof et al., 2009; Pan et al., 2010; Stösser et al., 2011; Mantyh, 2013; Schmidt, 2014). One such mediator is parathyroid hormone-related peptide (PTHrP), which is highly expressed in metastatic breast and prostate cancers (Iwamura et al., 1993; Soki et al., 2012). A recent study showed that the levels of the proteolytic fragment peptide PTHrP (12–48) are high in the plasma (range: 50 pg/μl to >200 pg/μl, with a mean of ~100 pg/μl or ~10 nM) of human patients with breast cancer bone metastasis vs. non-bone metastatic breast cancers (Washam et al., 2013). PTHrP selectively binds to the type-1 parathyroid hormone receptor (PTH1), but not the PTH2, and initiates a cascade of G protein-coupled receptor (GPCR)-mediated intracellular signaling (Hoare and Usdin, 2001). Both PTH1 and PTH2 are expressed in DRG neurons, with PTH1 being expressed on most DRG neurons, while PTH2 expression is largely confined to medium- and large-diameter myelinated nerves (Macica et al., 2006; Matsumoto et al., 2010; Mickle et al., 2015a).

We recently demonstrated that PTHrP can potentiate the activity of the nociceptive ion channel *t*ransient *r*eceptor *p*otential, family *v*anilloid, member-1 (TRPV1) and elicit peripheral pain hypersensitivity behaviors in mice (Mickle et al., 2015a). TRPV1 is predominantly expressed on peripheral nociceptors and can be activated by noxious heat, acidic pH, and a number of endogenous lipid mediators, as well as by exogenous algogens such as capsaicin (Caterina et al., 1997; Tominaga et al., 1998; Hwang et al., 2000; Loo et al., 2012; Mickle et al., 2015b). Initial characterization of *Trpv1*^−/−^ mice suggested that the TRPV1 channel is critical to the development of inflammatory heat hyperalgesia, without any influence on mechanical hypersensitivity (Caterina et al., 2000; Davis et al., 2000). However, several studies in the recent past have now suggested the involvement of TRPV1 in mechanical pain hypersensitivity in the context of a number of painful pathologies, such as inflammation, nerve injury, sickle cell disease and primary bone cancers (Ghilardi et al., 2005; McGaraughty et al., 2008; Shinoda et al., 2008; Hillery et al., 2011; Brenneis et al., 2013; Chung et al., 2015). Direct activation of TRPV1 by mechanical forces, such as those observed with peizo-driven polished blunt-tip glass pipets have not been shown. Rather, the slowly-adapting mechanical currents in mouse DRG neurons were shown to be completely blocked by a TRPA1 channel antagonist (Vilceanu and Stucky, 2010). A number of studies have suggested that protein kinase C (PKC) phosphorylation of TRPV1 channel is associated with the development of peripheral mechanical hypersensitivity in rodent models of painful pathologies, without any direct evidence that PKC could influence the channel and its contribution to mechanical sensitization (Lee et al., 2012; Chung et al., 2015; Wang et al., 2015). Overall, the prevailing view on the role of TRPV1 in mechanical pain sensitization is that neurogenic inflammation downstream of robust activation of this channel initiates a cascade of signaling, involving multiple pain-transducing ion channels and receptors, that culminates in the development of mechanical pain hypersensitivity (Patapoutian et al., 2009; Julius, 2013; Mickle et al., 2016; Gouin et al., 2017).

We recently found that both heat and mechanical hypersensitivity evoked by PTHrP were absent in *Trpv1^−/−^* mice (Mickle et al., 2015a). Conventionally, it is thought that mechano-transducing sensory ion channels, such as TRPA1 and TRPV4, contribute to the development of mechanical hypersensitivity (Kwan et al., 2006; Alessandri-Haber et al., 2008; Brierley et al., 2009; Ho et al., 2012; Nilius and Voets, 2013). Therefore, the aim of the current study was to make an unambiguous investigation of the contribution of nociceptive TRP channels to PTHrP-induced mechanical pain hypersensitivity. Our results suggest that TRPV1, and not TRPV4 or TRPA1 channel activity is required for the initiation of peripheral mechanical hypersensitivity induced by hindpaw injection of PTHrP in mice. We found that PTHrP perfusion leads to flow activation of TRPV1, which is dependent on PKC phosphorylation of the channel. Furthermore, peripheral TRPV1 channel activity significantly contributes to the maintenance of PTHrP-induced mechanical hypersensitivity.

## Materials and Methods

### Animals

All experiments were performed using adult (6–14 weeks old) mice housed in the University of Iowa and Washington University Animal facilities on a 12-h light/dark cycle with access to food and water *ad libitum*. All the procedures involving mice were approved by the Institutional Animal Care and Use Committees of The University of Iowa and Washington University in St. Louis, St. Louis, MO, USA and in strict accordance with the US National Institute of Health (NIH) Guide for the Care and Use of Laboratory Animals. Every effort was made to minimize the number of mice and their suffering in this study. C57BL/6J (B6-*WT*; Stock No: 000664), B6/129PF2/J (B6/129-*WT*; Stock No: 100903), B6/129PF2/J-*Trpa1*^−/−^ (B6/129-*Trpa1*^−/−^; Stock No: 006401), and C57BL/6J-*Trpv1*^−/−^ (B6-*Trpv1*^−/−^; Stock No: 003770) mice were purchased from Jackson Labs. The C57BL/6J-*Trpv4*^−/−^ mouse line was generated and generously provided by Dr. Wolfgang Liedtke (Liedtke and Friedman, 2003). Based on our previous findings on PTHrP-modulation of thermal and mechanical hypersensitivity in male vs. female mice (Mickle et al., 2015a), animals of both sexes were used in all behavioral, Ca^2+^ imaging and electrophysiological experiments. Intraplantar (i.pl.) injections were performed as described previously (Loo et al., 2012; Mickle et al., 2015a). Mice were manually restrained with the aid of a cloth such that the plantar surface of one hindpaw was exposed. A 10 μL volume was injected into the plantar surface of the hind paw via a 33-gauge stainless steel needle coupled to a Hamilton syringe. Mice were continuously monitored post-injection. Experimenters were blinded to mouse genotypes, saline/drug injection types and injection laterality during the conduct of experiments and data recordings.

### Chemicals and Reagents

Purified recombinant human/rodent collagenase and pronase were purchased from EMD Chemicals, and the Ca^2+^-sensitive dye Fura-2AM, pluronic acid, Lipofectamine-2000, Dulbecco’s modified-Eagle’s medium (DMEM), bovine serum and antibiotics for cell culture were purchased from Invitrogen—Thermo Fisher Scientific. PTHrP was purchased from Peprotech. AMG9810 (2E-N-(2,3-Dihydro-1,4-benzodioxin-6-yl)-3-(4-(1,1-dimethylethyl)phenyl)-2-Propenamide), capsaicin, GSK1016790A, poly-L-ornithine, and laminin were purchased from Merck-Millipore-Sigma. TNB-100 culture medium and Protein-lipid complex for neuron cultures were purchased from Biochrom-Merck-Millipore. 5’-Iodoresiniferotoxin (IRTX; 6,7-Deepoxy-6,7-didehydro-5-deoxy-21-dephenyl-21-(phenylmethyl)-daphnetoxin, 20-(4-hydroxy-5-iodo-3-methoxybenzeneacetate), Tetrodotoxin and Bisindolylmaleimide-I (BIM-1) were purchased from Tocris—R&D systems. All other chemicals used in this study were purchased from Merck-Millipore-Sigma, VWR and Thermo Fisher Scientific. The HEK293T cell line was purchased from American Type Culture Collection (ATCC), Manassas, VA, USA and routinely tested for mycoplasma contamination using the LookOut^®^ kit (Merck-Millipore-Sigma).

### Behavioral Assessment of Heat and Mechanical Hypersensitivity on Mouse Hindpaws

Mice were acclimated to the testing environments for 2 days prior to testing by placing them in the testing chambers for 30 min, two times a day separated by at least 1 h. Heat hypersensitivity was tested using Hargreaves’ method (IITC Life Sciences), as described previously (Loo et al., 2012; Mickle et al., 2015a). Briefly, mice were put in individual Plexiglas testing chambers placed on a glass plate maintained at thermo-neutral temperature (~30°C) for at least 30 min before testing. A focused high-intensity beam of light was illuminated onto the plantar surface of the hindpaw and the latency to paw withdrawal was recorded. For each time point, the paw withdrawal latency (PWL) was measured twice for both limbs and averaged for analysis.

To test mechanical sensitivity, mice were placed individually on a wire mesh platform covered by a Plexiglas box for 15 min before testing. Mechanical sensitivity was then measured counting paw withdrawals to applications of von Frey hair filaments (eight filaments; strength range 0.04–2 g; Stoelting Co.) applied to the plantar surface of the mouse hindpaw. Tests were performed starting with the lowest filament strength (0.04 g) and moving up to the filament with maximum strength (2 g). Each filament (in an ascending order of filament strength) was applied to each individual mouse hindpaw five times, and the number of paw withdrawal responses was recorded. To assess the changes in paw withdrawal response over the whole range of filaments for each testing period, the area under the curve (AUC) was calculated for each animal and the average AUC for each hindpaw and treatment group was calculated as detailed previously (Mickle et al., 2015a; Shepherd and Mohapatra, 2018). Baseline measurements were taken for both heat and mechanical sensitivity, and then after saline/drug injections for different durations. All behavioral experiments were performed on mice of individual genotypes (wherever mentioned), in two or more cohorts of animals.

### Primary Cultures of Mouse DRG Neurons

DRGs were isolated from adult B6-*WT*, B6-*Trpv1*^−/−^, B6-*Trpv4*^−/−^, B6/126-*WT* and B6/126-*Trpa1*^−/−^ mice, as described previously (Loo et al., 2012; Mickle et al., 2015a). Isolated ganglia (C4 to L5) were dissociated and digested with collagenase and pronase, and then plated onto poly-L-ornithine- and laminin-coated glass coverslips (for Ca^2+^ imaging or electrophysiology). Cells were incubated in culture media comprised a of 1:1 ratio of TNB media supplemented with protein-lipid complex, and DMEM with 10% fetal bovine serum, at 37°C in a 5% CO_2_ incubator for 2–3 days, before use in Ca^2+^ imaging and electrophysiological experiments.

### Functional Ca^2+^ Imaging

Ca^2+^ imaging experiments on cultured mouse DRG neurons were performed as described previously (Loo et al., 2012; Mickle et al., 2015a). The standard extracellular HEPES-buffered HBSS (HH buffer) contained (in mM) 140 NaCl, 5 KCl, 1.3 CaCl_2_, 0.4 MgSO_4_, 0.5 MgCl_2_, 0.4 KH_2_PO_4_, 0.6 NaHPO_4_, 3 NaHCO_3_, 10 glucose and 10 HEPES, pH 7.35 with NaOH (310 mOsm/kg with sucrose). Glass coverslips of mouse DRG neurons were incubated with 3 μM of Fura-2AM/pluronic acid for 20 min at room temperature (~22°C) prior to the experiment. The coverslips were washed in HH buffer following incubation to remove excess dye and then placed in the recording chamber mounted on the stage of an inverted Leica DMI6000B microscope, followed by at least a 5 min wash with HH buffer. Fluorescence was alternately excited at 340 and 380 nm (12 nm bandpass) using a Lambda LS Xenon lamp (Sutter Instrument) and a 10×/N.A 0.4 objective. Emitted fluorescence was collected at 510 nm using a Hamamatsu ORCA-100 CCD camera. Pairs of images were sampled at 1 Hz, background fluorescence was subtracted and the ratio of fluorescence (*F*_340_/*F*_380_) was calculated. Bath application of agonists (capsaicin, AITC or GSK1016790A) was made twice (15 s each for capsaicin and AITC; 30 s for GSK1016790A) in HH buffer, with a 3 min interval. The recording chamber was perfused with HH buffer for 1 min after the 1st agonist application, followed by vehicle or PTHrP in HH buffer for 2 min before the 2nd agonist application. Data were analyzed by calculating the ratio of the 2nd over 1st agonist-induced peak Ca^2+^ signal (*F*_340_/*F*_380_), in order to determine the magnitude of TRP channel sensitization. All Ca^2+^ imaging experiments were performed in ≥3 batches of DRG neuron cultures from each mouse genotype.

### Site-Directed Mutagenesis and Culture and Transfection of HEK293T Cells

The rat TRPV1 cDNA (in pcDNA3) was generously provided by Prof. David Julius. Site-directed mutagenesis was performed on rTRPV1-pcDNA3 to generate the phospho-disruptive alanine substitution at PKC phosphorylation sites, S502, T704 and S800 (rTRPV1-S502A/T704A/S800A or rTRPV1-TM as denoted in the figure), as described previously (Mohapatra et al., 2003; Loo et al., 2012). Mutations at only these three residues were confirmed by DNA sequencing of the full cDNA. Human embryonic kidney cells stably expressing the T-antigen (HEK293T) were cultured in DMEM (with Glutamax), 10% FBS and penicillin/streptomycin. Cells were co-transfected with plasmids containing eYFP-N1 (Clontech) and wild-type (WT) or PKC-triple mutant TRPV1, and/or YFP-tagged rat PTH1 (generously provided by Prof. Matthew Mahon) cDNAs using the Lipofectamine-2000™ reagent per the manufacturer’s instructions, as detailed previously (Mohapatra and Nau, 2003, 2005; Loo et al., 2012). Transfected cells were used for recordings 1–2 days post-transfection.

### Electrophysiology and Data Analysis

Voltage-clamp electrophysiological recordings in whole-cell mode were performed on cultured mouse DRG neurons and transfected HEK293T cells, as described previously (Loo et al., 2012; Mickle et al., 2015a). The pipette solution contained (in mM): 5 NaCl, 140 KCl, 1 CaCl_2_, 1 MgCl_2_, 10 HEPES, 5 EGTA and 3 Na-ATP, pH 7.3 with KOH. Cells were bathed in extracellular buffer containing (in mM) 140 NaCl, 5 KCl, 0.1 CaCl_2_, 1 MgCl_2_, 10 HEPES, 10 glucose, pH 7.3 with NaOH. For neuronal recordings 1 μM tetrodotoxin was added to the bath solution in order to block fast-activating Na_v_ currents. A low extracellular Ca^2+^ concentration was used to minimize the Ca^2+^-dependent desensitization of TRPV1 (Mohapatra et al., 2003). All agonists/drugs were diluted in the extracellular buffer and perfused locally onto the cell under recording with a flow rate of 2 ml/min, using individual channels of a gravity driven multiple-barrel perfusion system. Currents were recorded at room temperature (~22°C) with an Axopatch 200B patch-clamp amplifier connected to a Digidata 1440A data acquisition system (Molecular Devices). The holding potential was −70 mV, and the data were sampled at 2 kHz and filtered at 1 kHz using pClamp 10 software (Molecular Devices). Patch pipettes were pulled from borosilicate glass tubes and heat polished at the tip using a microforge (World Precision Instruments) to give a resistance of 2–5 MΩ when filled with the pipette solution. Clampfit 10 (Molecular Devices), Excel (Microsoft Co.), and Prism 7 (GraphPad Software) software were used for the analysis of currents and preparing traces/figures.

### Statistics

Data are presented as mean ± SEM. For behavioral and electrophysiological data, two-way ANOVA with multiple group comparisons and Bonferroni’s *post hoc* test were performed. *p* < 0.05 in each set of data comparisons was considered statistically significant. Ca^2+^ imaging data were analyzed using one-way ANOVA with Bonferroni’s *post hoc* test. All analysis was performed using GraphPad Prism 7.0 (GraphPad Software, Inc., La Jolla, CA, USA ).

## Results

### PTHrP-induced Heat and Mechanical Hypersensitivity Is Dependent on TRPV1, But Not TRPA1 or TRPV4

**Figure 1:**
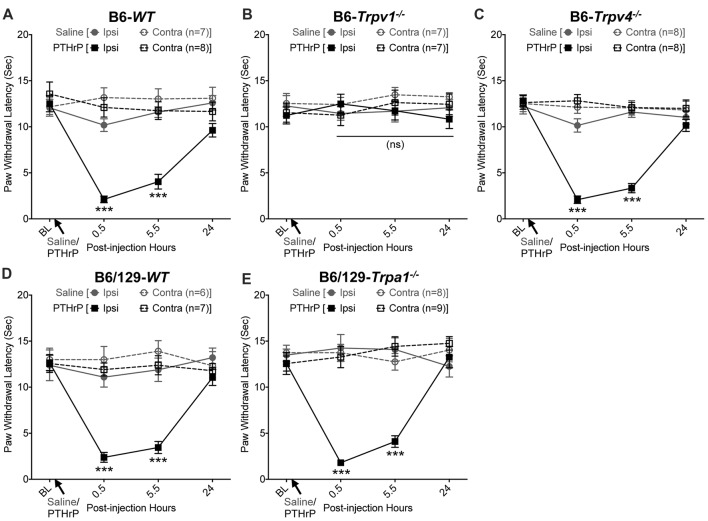
Parathyroid hormone-related peptide (PTHrP) induces heat hypersensitivity in mice, and is dependent on transient receptor potential channel family vanilloid, member-1 (TRPV1). PTHrP (1 μM in 10 μL, i.pl.) acutely caused a significant decrease in the paw withdrawal latency (PWL) to heat stimuli in the ipsilateral hindpaw of B6-wild-type (WT) mice, which persisted at 5.5 h after injection compared to saline injected controls **(A)**. This decrease in PWL by PTHrP injection was absent in B6-*Tprv1*^−/−^ mice **(B)**, but not in B6-*Trpv4*^−/−^ **(C)**. PTHrP-induced decrease in PWL was also observed in B6/129-*Trpa1*^−/−^ **(D)** to a similar extent as in B6/129-*WT* **(E)** and B6-*WT* mice **(A)**. Data are presented as mean ± SEM of hindpaw PWL. ***p < 0.001 and “ns”-not significant, two-way ANOVA with Bonferroni’s *post hoc* correction.

**Figure 2:**
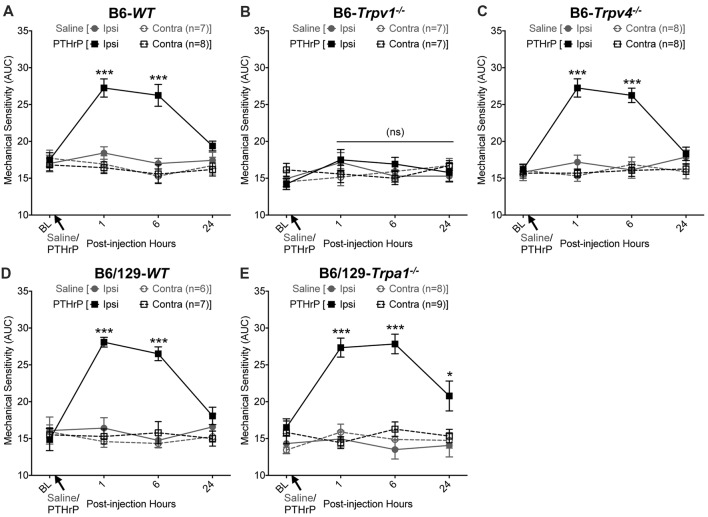
PTHrP induces mechanical hypersensitivity in mice, in a TRPV1-dependent manner. PTHrP injection (1 μM in 10 μL, i.pl.) led to a significant increase in mechanical sensitivity on mouse (B6-WT;** A**) ipsilateral hindpaw, as measured by area under the curve (AUC) of von Frey filament-response curve (see “Materials and Methods” section for details). Interestingly, PTHrP-induced hindpaw mechanical hypersensitivity was absent in B6-*Tprv1*^−/−^ mice **(B)**, but not in B6-*Trpv4*^−/−^ **(C)**, B6/129-*WT* **(D)**, and B6/129-*Trpa1*^−/−^ **(E)** mice. Data are presented as mean ± SEM of hindpaw AUC. *p < 0.05, ***p < 0.001 and “ns”-not significant, two-way ANOVA with Bonferroni’s *post hoc* correction.

We have previously shown that hindpaw administration of PTHrP induces robust thermal and mechanical hypersensitivity in mice with an early onset (0.5 h), and is persistent at 5.5 h post-injection (Mickle et al., 2015a). Our preliminary findings suggested the critical role of TRPV1 therein. We next verified the role of other TRP channels, such as TRPA1 and TRPV4, which have been suggested to play important roles in inflammatory pain hypersensitivity (Kwan et al., 2006; Alessandri-Haber et al., 2008; Patapoutian et al., 2009; Julius, 2013; Mickle et al., 2016). Hindpaw PTHrP injection (1 μM in 10 μL; i.pl.) led to the development of heat hypersensitivity in the ipsilateral hindpaws of WT (Figures 1A,D), *Trpv4*^−/−^ (Figure 1C) and *Trpa1*^−/−^ (Figure 1E) mice, but not *Trpv1*^−/−^ mice (Figure 1B). We must mention here that the PTHrP concentration used in these experiments (1 μM = 10 pmol in 10 μl injection volume) are based on a recent finding that the proteolytic fragment peptide PTHrP (12–48) are high in the plasma (range: 50 pg/μl to >200 pg/μl, with a mean of ~100 pg/μl or ~10 nM) of human patients with breast cancer bone metastasis vs. non-bone metastatic breast cancers (Washam et al., 2013). While it has been well established that TRPV1 plays a critical role in inflammatory heat hypersensitivity, its direct role in mechanical hypersensitivity remains a topic of debate (Caterina et al., 2000; Davis et al., 2000; Kwan et al., 2006; Patapoutian et al., 2009; Julius, 2013; Mickle et al., 2016). We next investigated if TRPV1 is also directly involved in PTHrP-induced mechanical hypersensitivity. PTHrP injection into mouse hindpaws led to robust mechanical hypersensitivity in WT mice (Figure 2A), whereas no significant alteration in hindpaw mechanical sensitivity was observed in *Trpv1*^−/−^ mice (Figure 2B). It has been shown that robust peripheral activation of TRPV1 initiates local neurogenic inflammation, which presumably leads to the activation of other mechano-transducing channels, such as TRPA1 and TRPV4, to induce mechanical pain hypersensitivity (Kwan et al., 2006; Alessandri-Haber et al., 2008; Brierley et al., 2009; Patapoutian et al., 2009; Julius, 2013; Mickle et al., 2016; Gouin et al., 2017). We therefore tested the role of TRPA1 and TRPV4 in PTHrP-induced mechanical hypersensitivity, presumably acting downstream of TRPV1. Interestingly, PTHrP-induced mechanical hypersensitivity was observed in *Trpv4*^−/−^ and *Trpa1*^−/−^ mice, to the same extent as seen with WT mice (Figures 2C–E). This observation suggests that TRPV4 and TRPA1 are not involved either upstream or downstream of TRPV1 activation to induce mechanical hypersensitivity upon PTHrP injection.

### PTHrP Sensitizes TRPV1-, But Not TRPA1- or TRPV4-mediated Ca^2+^ Influx

**Figure 3:**
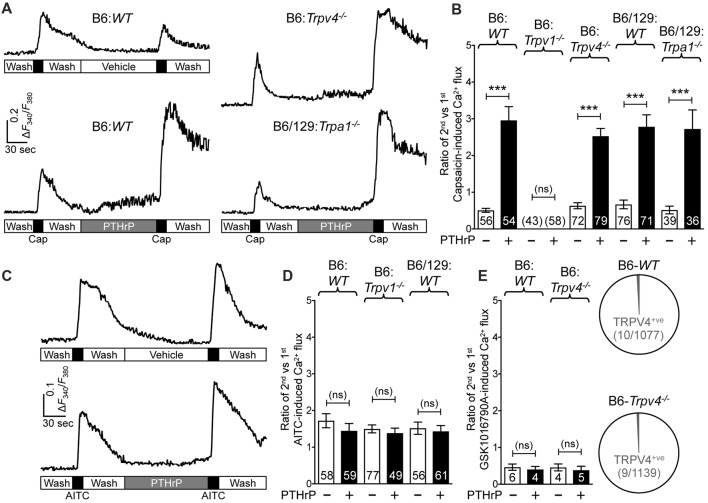
PTHrP potentiates Ca^2+^ influx through TRPV1, but not TRPA1 or TRPV4. **(A)** Representative Ca^2+^ imaging traces from cultured B6-*WT* (left panel traces), B6-*Trpv4*^−/−^ and B6/129-*Trpa1*^−/−^ (right panel traces) mouse DRG neurons with two successive application of capsaicin (50 nm, 15 s), with or without the perfusion of PTHrP (10 nM, 2 min) in between. **(B)** Quantification of the extent of PTHrP-induced potentiation of TRPV1-mediated Ca^2+^ influx by calculating the ratio of 2nd vs. 1st capsaicin-induced peak Ca^2+^ influx. PTHrP causes a significant potentiation of capsaicin-induced Ca^2+^ influx in cultured DRG neurons from B6-*WT*, B6-*Trpv4*^−/−^, B6/129-*WT* and B6/129-*Trpa1*^−/−^ mice, compared to respective vehicle controls. There was no quantifiable Ca^2+^ influx, and any change in the ratio of the 2nd vs. the 1st Ca^2+^ flux in cultured DRG neurons from *Trpv1*^−/−^ mice. **(C)** Representative Ca^2+^ imaging traces from cultured B6-*WT* mouse DRG neurons with two successive application of AITC (50 μm, 30 s), with or without PTHrP (10 nM, 2 min) perfusion in between. **(D)** PTHrP did not significantly alter AITC-mediated Ca^2+^ flux in cultured DRG neurons from B6-*WT*, B6-*Trpv1*^−/−^, and B6/129-*WT* mice, compared to respective vehicle controls. **(E)** Quantification of the ratio of 2nd vs. 1st peak GSK1016790A-induced (TRPV4 activator; 1 μm, 60 s) Ca^2+^ influx in cultured DRG neurons from B6-*WT* mice, with recordings made in a similar fashion as in **(A,C)**. PTHrP perfusion (10 nM, 2 min) did not cause any significant alteration in the ratio of 2nd vs. 1st peak GSK1016790A-induced Ca^2+^ influx in few cultured DRG neurons from both B6-*WT* and B6-*Trpv4*^−/−^ mice. Pie charts showing the number of neurons exhibiting any quantifiable GSK1016790A-induced Ca^2+^ influx in B6-*WT* (10 out of 1077 neurons) and B6-*Trpv4*^−/−^ (9 out of 1139 neurons) mouse cultured DRG neurons. Data in **(B,D,E)** are presented as mean ± SEM, and the individual group neuron numbers from ≥4 culture batches are indicated within the figure panels. ***p < 0.001 and “ns”-not significant, two-way ANOVA with Bonferroni’s *post hoc* correction.

We next wanted to determine if TRPA1 or TRPV4 channels could modulate TRPV1 meditated Ca^2+^ influx in response to PTHrP. As we have previously observed, PTHrP (10 nM, 2 min) could significantly potentiate capsaicin-mediated (50 nM, 15 s) Ca^2+^ influx in *WT* DRG neurons (Figures 3A,B). This concentration of PTHrP is based on a recent finding that the proteolytic fragment peptide PTHrP (12–48) are high in the plasma (mean = ~100 pg/μl or ~10 nM) of human patients with breast cancer bone metastasis vs. non-bone metastatic breast cancers (Washam et al., 2013). PTHrP potentiation of capsaicin-mediated Ca^2+^ influx was intact in *Trpa1^−/−^* and *Trpv4*^−/−^ DRG neurons, but was absent in *Trpv1*^−/−^ DRG neurons (Figures 3A,B). We also investigated if PTHrP could influence TRPA1- or TRPV4-mediated Ca^2+^ influx. Application of PTHrP (10 nM, 2 min) between two AITC applications (50 μM, 15 s) did not lead to any significant alteration in Ca^2+^ influx in DRG neurons cultured from *WT* and *Trpv1*^−/−^ mice, suggesting no direct influence of PTHrP on TRPA1 channel activity (Figures 3C,D). We next tested if PTHrP could modulate TRPV4-mediated Ca^2+^ influx. Using the TRPV4-specific agonist GSK1016790A (1 μM, 30 s), we did not observe any significant change in Ca^2+^ influx in mouse DRG neurons with PTHrP application (Figure 3E). Expression of TRPV4 in mouse DRG neurons has been debated lately, with studies showing no expression of functional TRPV4 channels (Alexander et al., 2013), and very low levels of *Trpv4* mRNAs (Girard et al., 2013; Goswami et al., 2014). Furthermore, it has been suggested that the highly potent and specific activator of TRPV4, GSK1016790A, induces mild-to-moderate levels of Ca^2+^ influx in 1%–2% of mouse DRG neurons from both WT and *Trpv4*^−/−^ mice (Alexander et al., 2013). In line with these observations, our analysis of >1000 cultured mouse DRG neurons from >5 WT and *Trpv4*^−/−^ mice each showed only 10 out of 1077 neurons in WT mice and 9 out of 1139 neurons in *Trpv4*^−/−^ mice showed any quantifiable Ca^2+^ influx, in response to GSK1016790A application (Figure 3E). Furthermore, PTHrP did not significantly alter GSK1016790A-mediated Ca^2+^ influx in those ~1% DRG neurons from both WT and *Trpv4*^−/−^ mice (Figure 3E). These findings suggest no functional TRPV4 expression in mouse DRG neurons, which also is in line with no involvement of TRPV4 in PTHrP-induced hindpaw heat/mechanical hypersensitivity (Figures 1C,  2C).

### PTHrP Induces TRPV1-dependent Inward Currents and Potentiation of Capsaicin-induced Currents in Mouse DRG Neurons and Transfected HEK293T Cells in a PKC Dependent Manner

**Figure 4:**
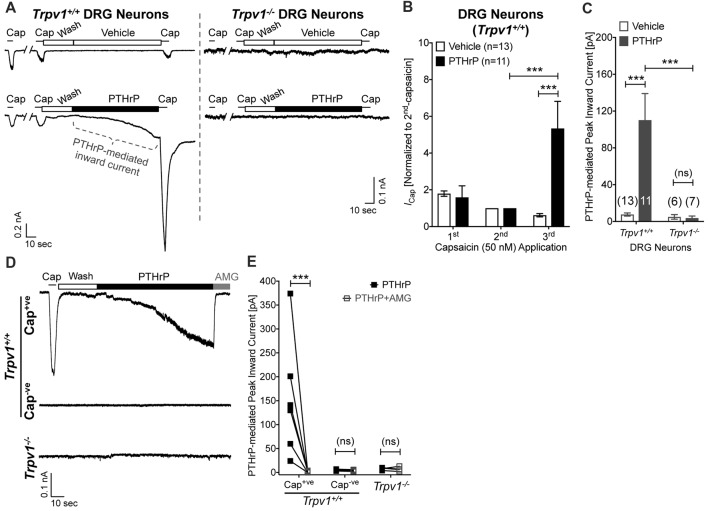
Potentiation and direct flow activation of TRPV1 currents in mouse DRG neurons by PTHrP. **(A)** Representative whole-cell current recordings from *Trpv1^+/+^* and *Trpv1*^−/−^ cultured DRG neurons showing three consecutive capsaicin (50 nM, ~5 s) application, with extracellular perfusion of vehicle or PTHrP (10 nM; 1.5 min) between 2nd and 3rd capsaicin application. **(B)** Quantification of the extent of PTHrP-induced potentiation of TRPV1 currents in cultured DRG neurons from *Trpv1*^+/+^ mice. **(C)** Peak inward currents mediated by on-cell perfusion of vehicle or PTHrP (10 nM; 1.5 min) in cultured DRG neurons from *Trpv1*^+/+^ and *Trpv1*^−/−^ mice. Data in **(B,C)** are presented as mean ± SEM, and the individual group cell numbers from culture batches of ≥4 mice are indicated within figure panels. **(D)** Representative whole-cell current recordings from capsaicin positive (Cap^+ve^) and negative (Cap^−ve^) cultured DRG neurons from *Trpv1*^+/+^ mice, as well as from DRG neurons from *Trpv1*^−/−^ mice showing sustained inward currents elicited by PTHrP (10 nM; 1.5 min) in Cap^+ve^ (50 nM, ~5 s) neurons only. This PTHrP-induced sustained inward currents can be completely blocked by the selective TRPV1 inhibitor AMG9810 (10 μM), as quantified in **(E)** ***p < 0.001 and “ns”-not significant, one-way ANOVA with Bonferroni’s *post hoc* correction.

Our previous study has shown potentiation of TRPV1 currents by PTHrP, which is largely dependent on Src signaling downstream of PTH1 activation (Mickle et al., 2015a). Interestingly, Src inhibitor administration led to attenuation of PTHrP-induced heat, but not mechanical hypersensitivity on mouse hindpaws (Mickle et al., 2015a). Since, TRPV1 is critically involved in PTHrP-induced mechanical hypersensitivity, we next investigated if PTHrP could directly influence TRPV1 channel activation in the absence of any exogenous agonist. Perfusion of PTHrP (10 nM, 1.5 min) in between two capsaicin applications (50 nM, ~5 s) led to significant potentiation of capsaicin currents in WT mouse DRG neurons (Figures 4A,B). Interestingly, PTHrP perfusion led to a steady increase in inward currents in WT capsaicin-positive mouse DRG neurons, which was absent in DRG neurons from *Trpv1^−/−^* mice (Figures 4A,C–E), indicating this current to be mediated by TRPV1. We further investigated if the PTHrP-induced inward currents in DRG neurons could also be contributed by channels other than TRPV1, following its opening and/or Ca^2+^ influx. Perfusion of the specific inhibitor of TRPV1, AMG9810 (10 μM), along with PTHrP rapidly and completely inhibited the inward current in its entirety (Figures 4D,E), indicating that PTHrP perfusion-induced sustained inward currents in mouse DRG neurons are contributed by TRPV1 channel only.

**Figure 5:**
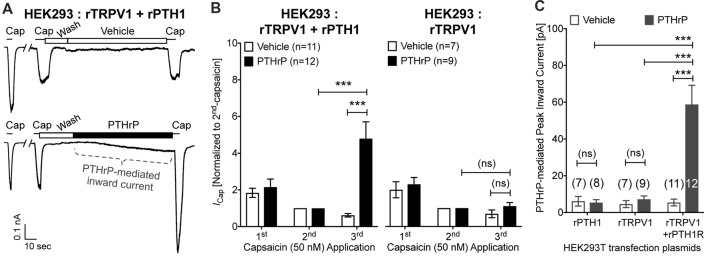
Potentiation and direct flow-activation of TRPV1 currents and its dependance on PTH1-PTHrP signaling in heterologous expression system. **(A)** Representative whole-cell current recordings from HEK293T cells transfected with plasmids containing rat TRPV1 (rTRPV1) and rat PTH1 (rPTH1) cDNAs showing three consecutive capsaicin (50 nM; ~5 s) application, with extracellular perfusion of vehicle or PTHrP (10 nM; 1.5 min) between 2nd and 3rd capsaicin application. **(B)** Quantification of the extent of PTHrP-induced potentiation of TRPV1 currents in HEK293T cells transfected with rTRPV1 and rPTH1 (left panel/group) or with rTRPV1 alone (right panel/group). **(C)** Peak inward currents mediated by on-cell perfusion of vehicle or PTHrP (10 nM; 1.5 min) in HEK293T cells transfected with rPTH1, or rTRPV1, or rTRPV1 and rPTH1. Data in **(B,C)** are presented as mean ± SEM, and the individual group cell numbers from ≥4 transfection culture batches are indicated within figure panels. ***p < 0.001 and “ns”-not significant, one-way ANOVA with Bonferroni’s *post hoc* correction.

In order to further confirm these observations, we utilized heterologous expression of TRPV1 and PTH1, alone or in combination in HEK293T cells. Perfusion of PTHrP (10 nM, 1.5 min) in between two capsaicin applications (50 nM, ~5 s) led to potentiation of TRPV1 currents in HEK293T cells expressing both TRPV1 and PTH1, but not in cells expressing TRPV1 alone (Figures 5A,B). Similar to our observation in DRG neurons, PTHrP perfusion led to a steady increase in inward currents in HEK293T cells expressing both TRPV1 and PTH1, but not in cells expressing TRPV1 alone or PTH1 alone (Figures 5A,C). These findings confirmed that PTHrP activation of PTH1 subsequently leads to TRPV1 channel activation without any exogenous activator.

**Figure 6:**
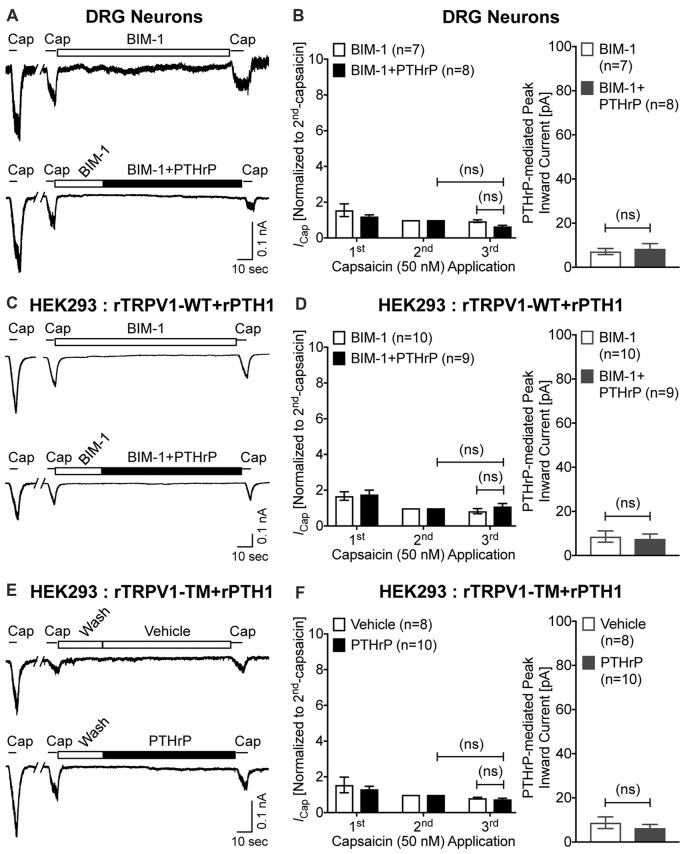
PTHrP-mediated potentiation and direct flow-activation of TRPV1 currents is dependent on protein kinase C (PKC) phosphorylation of the channel. **(A)** Representative whole-cell current recordings from cultured mouse DRG neurons showing three consecutive capsaicin (50 nM, ~5 s) application, with extracellular perfusion of a specific inhibitor of PKC, BIM-1 (0.5 μM, 0.5 min), followed by BIM-1 (0.5 μM) alone or along with PTHrP (10 nM; 1.5 min) between 2nd and 3rd capsaicin application. **(B)** Quantification of the extent of PTHrP-induced potentiation of TRPV1 currents (left), as well as PTHrP-induced peak inward currents (right) in mouse DRG neurons, with or without BIM-1 application. **(C)** Representative whole-cell current recordings from HEK293T cells transfected with plasmids containing WT rat TRPV1 (rTRPV1-WT) and rat PTH1 (rPTH1) cDNAs showing three consecutive capsaicin (50 nM; ~5 s) application, with extracellular perfusion of BIM-1 (0.5 μM, 0.5 min), followed by BIM-1 (0.5 μM) alone or along with PTHrP (10 nM; 1.5 min) between 2nd and 3rd capsaicin application. **(D)** Quantification of the extent of PTHrP-induced potentiation of TRPV1-WT currents (left), as well as PTHrP-induced peak inward currents (right) in HEK293T cells, with or without BIM-1 application. **(E)** Representative whole-cell current recordings from HEK293T cells transfected with plasmids containing rat TRPV1 with phospho-disruptive mutations at PKC phosphorylation sites, S502, T704 and S800 (rTRPV1-TM) and rat PTH1 (rPTH1) cDNAs showing three consecutive capsaicin (50 nM; ~5 s) application, with extracellular perfusion of vehicle or PTHrP (10 nM; 1.5 min) between 2nd and 3rd capsaicin application. **(F)** Quantification of the extent of PTHrP-induced potentiation of TRPV1-TM currents (left), as well as vehicle or PTHrP-induced peak inward currents (right) in HEK293T cells. Data in **(B,D,F)** are presented as mean ± SEM, and the individual group cell numbers from ≥4 mice and/or transfection culture batches are indicated within individual figure panels. “ns”-not significant, one-way ANOVA with Bonferroni’s *post hoc* correction.

Previous studies focusing on modulation of TRPV1 channel activation by PKC have shown that high concentrations of synthetic PKC activators, such as phorbol 12-myristate 13-acetate (PMA) and 12-O-tetradeconoylphorbol-13-acetate (TPA), could lead to TRPV1-mediated Ca^2+^ influx and channel activation in heterologous systems, in a PKC-dependent manner (Premkumar and Ahern, 2000; Vellani et al., 2001; Bhave et al., 2003). We next investigated if PTHrP-inward currents, as well as the potentiation of TRPV1 activation are dependent on PKC. Co-application of the PKC inhibitor BIM-1 (0.5 μM) completely attenuated both PTHrP-induced inward current and potentiation of TRPV1 channel activation in cultured mouse DRG neurons, as well as in HEK293T cells co-expressing WT rTRPV1 and rPTH1 (Figures 6A–D). Furthermore, the tripple Ala-substitution phospho-disruptive mutant TRPV1 channel at PKC phosphorylation sites S502, T704 and S800 (PKC-TM), when expressed in HEK293T cells along with rPTH1 failed to elicit any inward currents and potentiation of TRPV1 activation upon PTHrP perfusion (Figures 6E,F). These results suggest that PTHrP-PTH1-PKC activation could lead to constitutive and sustained activation of TRPV1 channel, without any other physico-chemical activator of the channel.

## Pharmacological Inhibition of Local TRPV1 Reduces PTHrP-induced Mechanical Hypersensitivity

**Figure 7:**
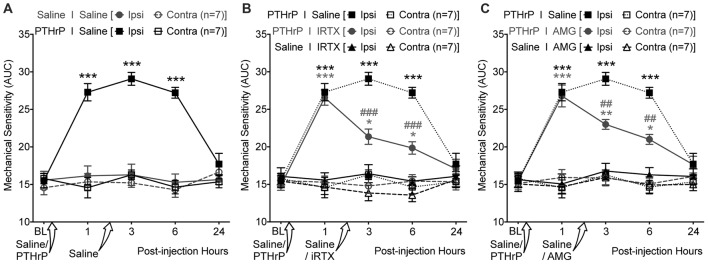
PTHrP-induced mechanical hypersensitivity could be acutely attenuated with TRPV1 antagonists.** (A)** PTHrP injection (1 μM in 10 μL; i.pl.) led to a significant increase in mechanical sensitivity of mouse (B6-*WT*) ipsilateral hindpaws, which persisted 6 h after injection (i.e., 3 h after injection of saline i.pl. at the same site). **(B)** Administration of TRPV1 antagonist (50 nM 5’-Iodoresiniferotoxin (IRTX) in 10 μL; i.pl.) 2 h post-PTHrP injection (1 μM in 10 μL; i.pl.) significantly attenuates hindpaw mechanical sensitivity in mice. Administration of IRTX (50 nM in 10 μL; i.pl.) 2 h post-saline injection (10 μL; i.pl.) did not influence hindpaw mechanical sensitivity in mice. **(C)** Administration of another TRPV1 antagonist (10 μM AMG8910 in 10 μL; i.pl.) 2 h post-PTHrP injection (1 μM in 10 μL; i.pl.) also led to significant attenuation of hindpaw mechanical sensitivity in mice. Administration of AMG9810 (10 μM in 10 μL; i.pl.) 2 h post-saline injection (10 μL; i.pl.) did not lead to any alteration in hindpaw mechanical sensitivity in mice. Data in all panels represent AUC of von Frey filament-response curves (see “Materials and Methods” section for details). All data are presented as mean ± SEM of hindpaw AUC. The AUC data for ipsilateral and contralateral hindpaws in PTHrP I Saline group from **(A)** are re-plotted in **(B,C)** as dotted lines for drug vs. saline group comparison and statistical analysis. *p < 0.05, **p < 0.01, and ***p < 0.001 vs. respective saline control/drug ipsilateral groups; ^##^*p* < 0.01 and ^###^*p* < 0.001 vs. respective PTHrP I Saline ipsilateral group; two-way ANOVA with Bonferroni’s *post hoc* correction.

We next tested if TRPV1 channel activity could be directly linked to PTHrP-induced mechanical hypersensitivity on mouse hindpaws. Local administration of a highly potent specific small molecule antagonist of TRPV1, IRTX (50 nM in 10 μL; i.pl.) 2 h post PTHrP injection (1 μM in 10 μL; i.pl.) led to significant attenuation of hindpaw mechanical hypersensitivity, as compared to saline-injected control (Figures 7A,B). Administration of another specific small molecule antagonist of TRPV1, AMG9810 (10 μM in 10 μL; i.pl.) 2 h post PTHrP injection (1 μM in 10 μL; i.pl.) also led to significant attenuation of hindpaw mechanical hypersensitivity, as compared to saline-injected control (Figure 7C). Administration of IRTX (50 nM in 10 μL; i.pl.) or AMG9810 (10 μM in 10 μL; i.pl.) 2 h post saline injection (10 μL; i.pl.) did not lead to any significant alteration in hindpaw mechanical sensitivity, similar to that in double saline-injected mice (Figures 7A–C). These results suggest that local TRPV1 activity is required in part for peripheral mechanical hypersensitivity induced by PTHrP.

## Discussion

Our study indicates that PTHrP elicits thermal and mechanical hypersensitivity in mice, both of which are dependent upon expression of functional TRPV1 channels. In addition, both PTHrP-induced heat and mechanical hypersensitivity are unaffected by genetic deletion of TRPV4 or TRPA1, suggesting that the development of heat, as well as mechanical hypersensitivity, are predominantly driven by TRPV1 activity. These behavioral observations are consistent with our *in vitro* Ca^2+^ imaging data, which again specifically implicate direct activation and modulation of TRPV1 in the potentiation of sensory neuron activity by PTHrP, and exclude a role for other mechano-transducing TRPA1 or TRPV4 channels in this process. Our electrophysiological analysis shows slow and sustained TRPV1 channel activation by PTHrP/PTH1 in the absence of any exogenous channel agonists in DRG neurons and in heterologous expression system. Furthermore, this current could be completely inhibited by a specific TRPV1 antagonist, thereby ruling out the contribution of other channels. Consistent with these observations, local administration of specific small molecule antagonists of TRPV1 attenuated PTHrP-induced mechanical hypersensitivity on mouse hindpaws, suggesting its involvement in peripheral mechanical pain sensitization.

PTHrP has been shown to be instrumental in breast and prostate cancer tumor growth and bone metastasis, and is enriched in the metastatic bone tumor microenvironment (Soki et al., 2012). A recent report showed that levels of proteolytic fragment of PTHrP are elevated in the plasma of breast cancer patients with bone metastasis vs. non-bone metastatic tumors, and could be utilized as a biomarker for bone-metastasized breast cancer (Washam et al., 2013). We have previously shown that it plays a critical role in nociceptor excitation and induction of pain hypersensitivity (Mickle et al., 2015a). Signaling downstream of the PTH1 receptor may therefore represent (along with local acidosis and other, pro-inflammatory mediators) a molecular and cellular mechanism underlying pain associated with metastatic bone cancer. TRPV1 has been shown to be expressed in sensory nerve fibers innervating hind limb bones (Ghilardi et al., 2005; Shepherd and Mohapatra, 2012). Prior studies have shown the involvement of TRPV1 in rodent models of primary bone cancer pain, and TRPV1 antagonists significantly attenuated primary bone cancer-related pain behaviors (Ghilardi et al., 2005; Shinoda et al., 2008; Pan et al., 2010). Together with our findings, it could be suggested that tumor microenvironment-enriched factors, such as PTHrP, could lead to constitutive activation of local nociceptive afferents, thereby constituting a tumor-nerve crosstalk mechanism underlying chronic pain.

We observed a lack of mechanical hypersensitivity following PTHrP administration in *Trpv1^−/−^* mice. It is a distinct possibility that one of the reasons for this failure of *Trpv1^−/−^* mice to respond to PTHrP is that neurogenic inflammation does not occur. In other words, the absence of functional TRPV1 channels precludes the release of neuropeptides and induction of pro-inflammatory processes downstream of TRPV1-mediated Ca^2+^ influx, thereby preventing the development of sustained mechanical hypersensitivity. Activation and/or modulation of TRPV4 and TRPA1 have previously been proposed to play an important role in the development of inflammatory mechanical hypersensitivity downstream of neurogenic inflammation (Kwan et al., 2006; Alessandri-Haber et al., 2008; Brierley et al., 2009; Patapoutian et al., 2009; Julius, 2013; Mickle et al., 2016; Gouin et al., 2017). However, our results indicate no involvement of TRPV4 and TRPA1 in PTHrP-induced mechanical hypersensitivity, rather TRPV1 serves as the critical channel, presumably through PTHrP/PTH1-mediated sustained activation of the channel. This is confirmed by our observation that PTHrP/PTH1-mediated sustained inward current in sensory neurons is mediated by TRPV1 in its entirety. Attenuation of PTHrP-induced mechanical hypersensitivity by the TRPV1 antagonists IRTX and AMG9810 is consistent with the hypothesis that TRPV1 is involved in mechanical hypersensitivity. However, the fact that TRPV1 antagonists are able to attenuate mechanical hypersensitivity after it has already developed also indicates that there is an ongoing requirement for TRPV1 function in the maintenance of mechanical hypersensitivity. It must be noted here that TRPV1 antagonists did not completely attenuate PTHrP-induced mechanical hypersensitivity. Therefore, further studies are required to better understand the downstream effects of TRPV1-mediated neurogenic inflammatory processes, and their relative contribution to the induction vs. the maintenance of heat and mechanical hypersensitivity.

The TRPV1-dependent current observed in DRG neurons induced by the application of PTHrP in the absence of any exogenous activator of TRPV1 suggests there could be constitutive activation of TRPV1 induced by PTH1 receptor signaling. Our findings show that both TRPV1 channel and PTH1 are both required for this current, without any contribution from other sensory channels. Furthermore, our results show that PTHrP-induced inward current is also dependent on PKC phosphorylation of TRPV1. Initial phenotypic reports of the TRPV1-null mouse appeared to rule out a role for TRPV1 in normal or pathological mechanotransduction (Caterina et al., 2000), but subsequent studies in inflammatory, nerve injury, muscle injury, sickle cell and sarcoma models have all implicated TRPV1 in mechanical hypersensitivity (McGaraughty et al., 2008; Shinoda et al., 2008; Pan et al., 2010; Hillery et al., 2011; Walder et al., 2012; Brenneis et al., 2013; Chung et al., 2015). We have previously shown that Src-mediated trafficking and potentiation of TRPV1 activity downstream of PTHrP increases spontaneous and capsaicin-evoked action potential firing in DRG neurons, suggesting that similar signaling might also underlie this phenomenon. However, Src inhibition was unable to attenuate PTHrP-induced mechanical hypersensitivity on mouse hindpaws (Mickle et al., 2015a). It is plausible that PKC activation plays a predominant role in constitutive mechanical activation of TRPV1 channel to flow pressure, which is in part supported by our results in the present study. The exogenous PKC activators such as TPA and PMA have previously been shown to activate TRPV1 channel under heterologous expression system (Premkumar and Ahern, 2000; Vellani et al., 2001; Bhave et al., 2003). Therefore, future in-depth investigations are warranted to understand the structural and biophysical mechanisms underlying such constitutive activation of PKC-phosphorylated TRPV1 channel. However, it could be possible that other as-yet unidentified mediator(s) downstream of PTH1 activation could serve as an intracellular agonist for TRPV1 channel activation, which also needs to be explored in detail.

TRPA1 has been shown to mediate mechanical hypersensitivity in some contexts, such as inflammatory conditions and neuropathic pain induced by experimental nerve injury and chemotherapeutic drugs (Kwan et al., 2006; Brierley et al., 2009; Patapoutian et al., 2009; Julius, 2013; Mickle et al., 2016; Trevisan et al., 2016; Gouin et al., 2017). However, we find no evidence to support a role of TRPA1 in PTHrP-induced heat and mechanical hypersensitivity. TRPV4 has been reported to be activated by osmotic changes and mechanical forces such as shear stress (Alessandri-Haber et al., 2003; Köhler et al., 2006; Ho et al., 2012), but our data are consistent with prior observations that functional TRPV4 channels are not expressed on DRG neurons (Alexander et al., 2013), suggesting no involvement of this channel in PTHrP-induced mechanical hypersensitivity. A number of reports have shown that TRPV4 and TRPA1 channel activity could be modulated by PKC signaling (Cao et al., 2009; Meotti et al., 2017), although we did not observe that in our experiments with PTHrP. As such, our study did not detect functional expression of TRPV4 in mouse DRG neurons, similar to other’s findings (Alexander et al., 2013). TRPA1 modulation downstream of bradykinin B1 receptor-mediated PKC activation has been shown to contribute to mechanical hypersensitivity (Meotti et al., 2017). With regard to absence of TRPA1 modulation by PTHrP-PKC in our study, it could be speculated that PTH1 and TRPV1 are co-expressed in specialized local signaling complexes, which provides PKC-mediated channel modulatory signal to TRPV1 only. Such a possibility needs to be experimentally verified, with the identification of individual components of such signaling complexes.

From prior reports and our present finding, we cannot exclude the possibility that TRPV1 drives the development and maintenance of mechanical hypersensitivity via interaction(s) with channels other than TRPA1 and TRPV4. For example, prior studies have reported interaction of TRPV1 with NMDA receptors and with lysophosphatidic acid receptors via a PKC-mediated mechanism to mediate peripheral mechanical hypersensitivity in the context of muscle pain and bone cancer pain (Pan et al., 2010; Lee et al., 2012). Alternatively, PKC and Src, both of which are activated downstream of PTH1 activation (Mickle et al., 2015a), are known to modulate other ion channels expressed in sensory neurons, such as ASIC3, Na_v_1.7 and Na_v_1.8 (Amir et al., 2006). Further studies will attempt to address these potential interactions. Another set of mechano-transducing channels have been described more recently, the Piezo channels (Coste et al., 2012). The role of Piezo1 and Piezo2 channels in the development of inflammatory, cancer and/or neuropathic pain conditions remains to be explored in detail. One study showed that Piezo2 channel activation to mechanical touch-force could be significantly potentiated by bradykinin-mediated activation of PKA and PKC (Dubin et al., 2012). Subsequent studies have now shown that Piezo2 is not involved in mechanical transduction in nociceptive neurons, rather are critical in touch sensation mediated by proprioceptive sensory neurons (Ranade et al., 2014; Woo et al., 2014). Interestingly, it has been recently shown that TRPV1 channel activation in DRG neurons inhibits Piezo1- and Piezo2-mediated mechanosensitive currents by depleting phosphatidylinositol 4,5-bisphosphate and phosphatidylinositol 4-phosphate from the plasma membrane through activation of the Ca^2+^-induced phospholipase Cδ (Altier, 2015; Borbiro et al., 2015). Taken together, the role of nociceptive channels other than TRPV1 in PTHrP-mediated mechanical hypersensitivity seems secondary.

Understanding the extremely diverse and context-dependent functions of ion channels in sensory neurons that detect and transduce painful stimuli continues to represent a formidable challenge. Nonetheless, furthering our understanding of interactions between pathological mediators such as PTHrP, the signaling mechanisms they employ and their physico-chemical interactions with sensory ion channels will increase the likelihood of developing more efficacious analgesic approaches for debilitating pain states, such as bone-metastasized cancer.

## Author Contributions

AJS, ADM and SK contributed equally to this work. AJS, ADM and DPM performed behavioral experiments and designed the study. SK and AJS performed Ca^2+^ imaging experiments. ADM and DPM performed electrophysiology experiments. HH provided critical reagents and mouse models. All authors contributed to data analysis and manuscript writing.

## Conflict of Interest Statement

The authors declare that the research was conducted in the absence of any commercial or financial relationships that could be construed as a potential conflict of interest.
